# Biochemical Analysis of DNA Polymerase η Fidelity in the Presence of Replication Protein A

**DOI:** 10.1371/journal.pone.0097382

**Published:** 2014-05-13

**Authors:** Samuel C. Suarez, Shannon M. Toffton, Scott D. McCulloch

**Affiliations:** 1 Department of Biological Sciences, North Carolina State University, Raleigh, North Carolina, United States of America; 2 Center for Human Health and the Environment, North Carolina State University, Raleigh, North Carolina, United States of America; University of Miami Miller School of Medicine, United States of America

## Abstract

DNA polymerase η (pol η) synthesizes across from damaged DNA templates in order to prevent deleterious consequences like replication fork collapse and double-strand breaks. This process, termed translesion synthesis (TLS), is an overall positive for the cell, as cells deficient in pol η display higher mutation rates. This outcome occurs despite the fact that the *in vitro* fidelity of bypass by pol η alone is moderate to low, depending on the lesion being copied. One possible means of increasing the fidelity of pol η is interaction with replication accessory proteins present at the replication fork. We have previously utilized a bacteriophage based screening system to measure the fidelity of bypass using purified proteins. Here we report on the fidelity effects of a single stranded binding protein, replication protein A (RPA), when copying the oxidative lesion 7,8-dihydro-8-oxo-guanine(8-oxoG) and the UV-induced *cis-syn* thymine-thymine cyclobutane pyrimidine dimer (T-T CPD). We observed no change in fidelity dependent on RPA when copying these damaged templates. This result is consistent in multiple position contexts. We previously identified single amino acid substitution mutants of pol η that have specific effects on fidelity when copying both damaged and undamaged templates. In order to confirm our results, we examined the Q38A and Y52E mutants in the same full-length construct. We again observed no difference when RPA was added to the bypass reaction, with the mutant forms of pol η displaying similar fidelity regardless of RPA status. We do, however, observe some slight effects when copying undamaged DNA, similar to those we have described previously. Our results indicate that RPA by itself does not affect pol η dependent lesion bypass fidelity when copying either 8-oxoG or T-T CPD lesions.

## Introduction

DNA replication in the presence of damaged bases requires specialized DNA polymerases in order to prevent more deleterious consequences caused by replicative polymerase stalling [1]. One member of the Y-family, DNA polymerase η (pol η), replicates past UV light induced DNA lesions like *cis-syn* thymine-thymine cyclobutane pyrimidine dimers (T-T CPD) with similar fidelity to that of copying undamaged DNA but with much higher efficiency [2]. Pol η and other Y-family polymerases demonstrate much lower fidelity than replicative polymerases when copying undamaged DNA, and their access to DNA is likely tightly controlled by mechanisms that include (but are not limited to) mono-ubiquitylation of the sliding clamp PCNA [3]. In contrast with the similar error rates of 1 error in ∼30 insertions when copying T-T CPD and undamaged DNA [2], [4], [5], human pol η copies the ubiquitous oxidative lesion, 7,8-dihydro-8-oxo-guanine (8-oxoG) with an error rate approaching 1 in 2, or 50% [6]–[8]. Despite this very low fidelity, pol η copies past 8-oxoG more efficiently than it copies undamaged DNA of the same sequence [8]–[10]. This contrasts with the fidelity of *S. cerevisiae* pol η, which copies 8-oxoG with much higher fidelity [8], [11]. Despite these *in vitro* derived bypass error rates, cells deficient in pol η display higher mutation rates when transfected with DNA treated with methylene blue plus visible light, which preferentially creates 8-oxoG lesions in DNA [12]. A similar increase in mutations is seen when XPV cells that are deficient in pol η are exposed to UV light [13], [14]. Many possibilities exist to explain this paradox of a polymerase that creates mutations, but whose presence is an overall positive for the cell.

One explanation of how moderate-to-low fidelity bypass by pol η still allows a reduction of mutagenesis is by modulation of pol η fidelity by interaction with one or more of the many replication accessory proteins present at a replication fork. However, a long record of DNA replication fidelity studies have shown a less than clear record of interactions with replication accessory proteins that increase polymerase fidelity. When examining the bacteriophage polymerases from RB69, T4 and T7, there is little evidence that replication accessory proteins have large effects on polymerase fidelity [15]–[18]. Some small changes are observed when examining the effect of the processivity clamp on *E. coli* Pol III fidelity, but not Pol IV, a lesion bypass polymerase [19], [20]. *Thermus thermophilius* single stranded binding protein (SSB) slightly increases the fidelity of the exonuclease deficient *T. thermophilius* polymerase when PCR is performed on the pUC19 plasmid [21], although an indirect role in protecting the DNA substrate cannot be ruled out in this report. When examining eukaryotic polymerases, the evidence is just as varied. Polymerase α from *S.* *cerevisiae* exhibits similar mutation frequencies when examining 3 SSBs from yeast, and one of those SSBs resulted in slightly reduced single base deletions when copying 3–5 reiterated nucleotides [22]. Yeast pol α also shows no difference in base substitution fidelity or single base deletion mutant frequency when adding yeast RPA [23]. Pol α from HeLa cell extracts exhibits a ∼5-fold reduction in mutation frequencies when copying shuttle vectors in response to addition of human RPA [24]. Calf thymus pol α exhibits reduced terminal misincorporation at pol α pause sites with the addition of RPA [25], while also reducing misincorporation efficiency about between 5- and 6-fold [26]. Polymerases that accomplish the majority of replication show other effects. PCNA actually increases calf thymus pol δ misincorporation [27], but it decreases *S. cerevisiae* pol δ fidelity ∼2-fold [28]. This contrasts with a report by Fortune *et al* reporting PCNA and RPA do not decrease base substitutions by *S. cerevisiae* pol δ, but PCNA and RPA reduce deletions ∼10-fold individually and ≥90-fold together [29]. While these reports suggest that replication accessory proteins do have some ability to alter the fidelities of polymerases, they do not speak to the synthesis across from damaged DNA templates, which occurs by polymerases that are significantly different than the replicative polymerases.

Reports examining eukaryotic polymerases involved in TLS have also been varied. The fidelity of the B-family polymerase ζ from *S. cerevisiae*, which plays a role primarily in extending mismatched primer termini, is not affected by the combination of replication factor C (RFC; the 5-subunit PCNA loading complex), PCNA, and RPA [30]. This is similar to results from *S. cerevisiae* pol η when copying both T-T CPD or 8-oxoG in the presence of RPA, RFC, and PCNA [8], [31]. Both of these reports utilize an assay that requires multiple insertion and extension events past damaged templates. Both reports show little effect of these proteins on bypass fidelity [30], [31]. These reports contrast with work by Maga *et al* who reported a 6-fold reduction in human pol η misincorporation of dATP when adding just PCNA to a single nucleotide incorporation experiment with 8-oxoG in the template. This same misincorporation event is reduced 21-fold when adding both RPA and PCNA [7]. This report utilized polymerase excess over substrate DNA, measured only single nucleotide insertion kinetics, lacked RFC that could load PCNA onto primer termini, and also lacked any means of blocking PCNA from migrating off the DNA ends. While this is a very interesting result, the results are for PCNA alone as well as PCNA and RPA together, but not RPA alone.

A point that is often overlooked in the discussion of pol η bypass as “error-free” is that *S. cerevisiae* and human pol η differ in 8-oxoG bypass fidelity [6], [8], [11], but share similar T-T CPD bypass and undamaged DNA fidelity [2], [4], [5], [32], [33]. Taking into account previous reports [7], [8], we wished to determine the contribution of RPA to the fidelity of bypass by human pol η across from 8-oxoG and T-T CPD. We reasoned that the very low fidelity of 8-oxoG bypass by human pol η compared to yeast pol η (∼50% vs ∼5% error rate, respectively) could mean that accessory proteins do affect the human protein, despite their apparent lack of ability to affect yeast pol η. Here we utilized a well-described system that requires both insertion(s) across from the damaged nucleotides as well as extension beyond the lesion by polymerase. Extensive use of this assay and template sequence previously using only polymerase allows us to make a direct comparison of the ability of RPA to modify the fidelity of lesion bypass by human pol η.

## Materials and Methods

### DNA Oligomer Sequences

Oligonucleotide primers were purchased from Integrated DNA Technologies, Inc (Coralville, IA). Damaged and undamaged templates were purchased from Midland Certified Reagent Co. (Midland, TX). Substrates used for the lesion bypass fidelity assay were as follows. Template sequence is stated. Underlined portions indicate primer annealing. Bold **XX** indicate positions of T-T CPD. Bold **Y** indicates either undamaged G or 8-oxoG. Primers were purchased with cy5 5′ end labeling from IDT. 75 mer templates: 5′-biotin-AGGAAACAGCTATGACCATGATTACGAATTCCAGCTCGGTACCGGGTTA**Y**CCTTTGGAGTCGACCTGCAGAAATT-biotin. 5′-biotin-AGGAAACAGCTATGACCATGATTACGAATTCCAGCTCGGTACCGGG**XX**AGCCTTTGGAGTCGACCTGCAGAAATT-biotin. 75 mer upstream: 5′-biotin-CCAGCTCGGTACCGGGTTA**Y**CCTTTGGAGTCGACCTGCAGAAATTCACTGGCCGTCGTTTTACAACGTCGTGACT-biotin. 45 mer: 5′-CCAGCTCGGTACCGGGTTA**Y**CCTTTGGAGTCGACCTGCAGAAATT. Templates and primers were resuspended in ultrapure H_2_O. Substrates were created by mixing primer (5 µM final concentration) with 1.1× molar equivalent template in 25 mM Tris pH 8.0 and 100 mM NaCl, heating to 85°C for 5 minutes and then cooling to room temperature over 3 hours, protected from light.

### Recombinant Proteins

#### Pol η

The expression vector for n-terminally 6xHis-tagged human pol η (pJM879) and *E. coli* expression strain (BL21 [DE3] derivative RW644) was graciously provided by Dr. Roger Woodgate (NICHHD). Expression and purification was modified from a previously published protocol [34]. RW644 cells were transformed with pJM879 vector using electroporation and plated onto LB media containing 30 µg/mL kanamycin. Isolated colonies were picked and used to inoculate a 12 mL culture of LB with kanamycin. This culture was grown to saturation overnight at 37°C with constant shaking and used to inoculate 1L LB with kanamycin. Cultures were grown for 5 hours at 37°C with constant shaking, after which, cells were centrifuged, washed with PBS, and resuspended in PBS in a volume equal to that of the cell pellet. Resuspended cells were placed drop wise into liquid N_2_, freezing them into 3–5 mm spheres, which were pooled and stored at −80°C until use. Cell spheres were lysed using a SPEX Sample Prep 6870 Freezer/Mill. Cells were cooled in liquid N_2_ for 10 minutes, and then subjected to 6 cycles of 1 minute grinding (10 impacts per second) followed by 1 minute rest. The resulting lysed cell powder was resuspended in lysis buffer containing 50 mM Tris pH 7.5, 300 mM NaCl, 20 mM imidazole, 10 mM β-mercaptoethanol, and 10% glycerol and was supplemented with 0.2 mM PMSF and Roche Complete Protease inhibitor tablets (1 per 50 mL volume). The crude cell lysate was sonicated (Branson 250 sonifier; output-2, duty cycle 50%) 12–16 times for 30 seconds with one minute on ice in between cycles. Cell lysate was centrifuged for 30 minutes at 40,000 g, 4°C and the soluble fraction was passed through a Nalgene filter unit (.45 µM). The filtered lysate was applied to a 5 mL HiTrap Q FF (GE Life Sciences) and 5 mL HiTrap Chelating HP column (GE Life Sciences) charged with NiSO_4_, connected in sequence, and equilibrated in lysis buffer using an AKTA Purifier (GE Life Sciences). After loading, the HiTrap Q FF was removed and the remaining chelating column was washed with 5 column volumes Wash 1 (50 mM Tris pH 7.5, 10% glycerol, 1 M NaCl, 20 mM imidazole, and 10 mM β-mercaptoethanol). The column was then washed with 5 column volumes Wash 2 (10 mM Na-Pi pH 7.7, 300 mM NaCl, 10% glycerol, 20 mM imidazole, and 10 mM β-mercaptoethanol). The protein was eluted with a step gradient (Wash 2– Buffer H) consisting of 4 column volumes at 25% Buffer H followed by 6 column volumes of 100% Buffer H (10 mM Na-Pi pH 7.7, 300 mM NaCl, 10% glycerol, 200 mM imidazole, and 10 mM β-mercaptoethanol). Fractions with highest enrichment for pol η (as determined by SYPRO Red-stained SDS-PAGE) were pooled and loaded directly to a 5 mL BioRad Hydroxyapatite (Bio-Scale Mini CHT Type I) column. The column was washed with 5 column volumes of Buffer H and then an 8 column volume gradient of 10–125 mM sodium phosphate in Buffer H was applied, followed by 2 column volumes of 200 mM sodium phosphate in Buffer H. Fractions with the highest enrichment of pol η were buffer exchanged against Buffer M (20 mM NaPi pH 7.3, 10% glycerol, 100 mM NaCl, and 10 mM β-mercaptoethanol) using an Amicon Ultra-15 centrifugal filter unit (30,000 NMWL) (EMD Millipore). The sample was then applied to a 1 mL Mono S 5/50 GL column (GE Life Sciences). The column was washed with 8 column volumes of Buffer M, then with Buffer M containing 200 mM NaCl. Protein was eluted using a gradient of 200–600 mM NaCl in Buffer M. Peak fractions were flash frozen in liquid N_2_ in aliquots and stored at −80°C until further use.

Single amino acid substitution mutants were produced by mutating the pJM879 vector using the Agilent Technologies Quikchange II XL site directed mutagenesis kit according to manufacturer instructions. Primers were purchased from and synthesized by IDT. Primers, with codon changes underlined, were as follows: Q38A-F; 5′-TAAACCGTGCGCGGTTGTCGCATATAAAAGCTGGAAAGGG, Q38A-R; 5′-CCCTTTCCAGCTTTTATATGCGACAACCGCGCACGGTTTA, Y52E-F; 5′-GGTGCCATTATCGCAGTTTCTGAGGAAGCGCGCGCCTT, Y52E-R; 5′-AGGGCGCGCGCTTCCTCAGAAACTGCGATAATGCCACC.

C-terminal 6xHis-tagged truncated human pol η (aa 1–511) was purified as previously described in [9] and [10].

#### RPA

The pTYB-RPA vector encoding human RPA was graciously provided by Dr Yue Zou (East Tennessee State University). Purification of the chitin binding protein-intein-RPA70 fusion protein and RPA32, RPA14 subunits was purified as described below, a modification of a previously published report [35].

BL 21 [DE3] *E. coli* cells were transformed by electroporation and plated onto LB media with 100 µg/mL ampicillin and 50 µg/mL chloramphenicol. An isolated colony was used to inoculate 100 mL 2XYT containing 100 µg/mL ampicillin and 50 µg/mL chloramphenicol. Cultures were grown to saturation overnight with shaking at 37°C. 20 mL of saturated cultures were used to inoculate each of 4×1 L 2XYT media with ampicillin and chloramphenicol and were grown to an OD_600_ of ∼0.6. IPTG was added to a final concentration of 1 mM and cultures were grown an additional 3 hours at 25°C, after which cells were harvested by centrifugation, washed with PBS, pooled, and resuspended in a volume equal to that of the cell pellet. Resuspended cells were placed drop wise into liquid N_2_ and ground into powder as described above. The resulting lysed cell powder was resuspended in buffer A (20 mM Tris-Cl pH 8.0, 1 M NaCl.1 mM EDTA.1% Triton X-100, 1 mM DTT, 10% glycerol, 0.2 mM PMSF and Roche Complete Protease inhibitor tablets [1 per 50 mL volume]). Cell lysate was sonicated as described above and was centrifuged for 30 min at 15,000 g, 4°C. 5 mL of chitin beads were equilibrated in buffer A and added to the cleared lysate. This mixture was rotated for 30 minutes at 4° and applied to an empty BioRad Econo-Pac chromatography column (1.5×12 cm, 30 mL total volume). Flow was by gravity only. Resin was washed with 100 mL of buffer B (20 mM Tris-Cl pH 8.0, 800 mM NaSCN, 0.25 mM EDTA, 0.01% NP-40, 1 mM DTT, 10% glycerol). The resin was flushed with 25 mL cleave buffer (20 mM Tris-Cl pH 8.0, 500 mM NaCl, 0.1 mM EDTA, 10% glycerol) with 30 mM DTT, sealed, and stored at 4°C overnight. Protein was eluted with 20 mL cleave buffer containing only 1 mM DTT, and was collected in 1 mL aliquots. Selected peak fractions (as determined by SDS-PAGE) were pooled and buffer exchanged against buffer C (25 mM HEPES-OH pH 7.8.25 mM EDTA, 1 mM DTT, and 10% glycerol) with 50 mM KCl using an Amicon Ultra-15 centrifugal filter unit (10,000 NMWL) (EMD Millipore). The sample was then applied to a 1 mL Mono Q 5/50 GL column (GE Life Sciences) using an AKTA purifier (GE Life Sciences), and was washed with 20 mL buffer C with 50 mM KCl. Protein was eluted using a 30 mL gradient from 50 to 500 mM KCl in buffer C. Peak fractions (as determined by SDS-PAGE) were buffer exchanged to buffer C with 150 mM KCl by the same procedure as before. Buffer exchanged sample was flash frozen in liquid N_2_ and stored in aliquots at −80°C until use.

### Lesion Bypass Fidelity Assay

Cell strains, bacteriophage reagents, and protocol have been previously described [36]. All reactions were performed in 40 mM Tris pH 8.0, 250 µg/mL BSA, 10 mM DTT, 10 mM MgCl_2_, 60 mM KCl, and 1.25% glycerol. Reactions were supplemented with 100 µM final concentration of each dNTP. Restriction enzymes and nucleotides were purchased from New England Biolabs (Ipswich, MA). Reaction volumes for the lesion bypass assay were 50 µL. Reactions contained 10 pmoles substrate, 10 pmoles polymerase, and 50 pmoles RPA. RPA was added first and pre-incubated at 37°C for 30 minutes and synthesis was initiated by addition of polymerase. Reactions were incubated at 37°C for an additional 30 minutes. Reactions were stopped with the addition of 2 µL of 500 mM EDTA, and were processed according to previously described protocol [36]. After recovery, newly synthesized oligos were annealed to gapped M13 mp18 bacteriophage DNA (10–25 fold excess oligo over phage). Annealed gap DNA was transformed into MC1061 cells as previously described and plaque phenotype and numbers were counted. Dark blue plaques were amplified using Templiphi (GE Life Sciences) according to manufacturer protocol and resulting DNA was sequenced by Genewiz (Research Triangle Park, NC). Error rates were calculated as previously described [36].

### DNA Binding

DNA binding experiments were performed in 40 mM Tris pH 8.0, 250 µg/mL BSA, 10 mM DTT, 10 mM MgCl_2_, 60 mM KCl, and 1.25% glycerol. Reactions containing RPA were incubated at 37°C for 30 minutes. Reactions with RPA and polymerase were incubated at 37° for 30 minutes after the addition of RPA, and for an additional 5 minutes at 37° upon the addition of polymerase. Reactions with RPA, polymerase, and dNTPs were incubated at 37° for 30 minutes after the addition of RPA, 5 minutes at 37°C upon the addition of polymerase, and for an additional 30 minutes at 37°C with the addition of 100 µM final concentration of each dNTP. All reactions were stopped by the addition of 0.5 volumes of ice-cold 40% glycerol. The sample was placed on ice and loaded into a 6% acrylamide (19∶1 acrylamide:*bis*-acrylamide) gel. Gels were imaged with a Storm 865 imager (GE Life Sciences).

## Results and Discussion

There are conflicting reports [7], [8] on the ability of the replication accessory protein RPA to increase the fidelity of polymerase η during bypass of an 8-oxoG. We have previously shown, using an assay that requires multiple incorporations of all four dNTPs, that bypass of 8-oxoG by human pol η occurs with very low fidelity, with dATP being stably misincorporated ∼50% of the time [8]. We also demonstrated that the addition of RPA, RFC and PCNA had no effect on the fidelity using the yeast forms of these proteins. However, Maga *et al* have reported that addition of RPA and PCNA greatly increase the incorporation of dCTP by both human pol η and pol λ [7]. Possible reasons for these conflicting reports include the use of single nucleotide insertion assays versus our assay that utilizes all four nucleotides in competition, as well as the possibility that yeast and human pol η are different in this respect.

In order to directly test these possible explanations, we used an assay that uses *in vitro* DNA synthesis coupled with color based M13 phage plaque screening to measure the fidelity of human pol η in the absence and presence of human RPA when it bypasses 8-oxo-G lesions. We first determined the fidelity of pol η in the presence of RPA when copying 8-oxo-G using a truncated protein that contains the first 511 amino acids, including the catalytic core of the polymerase [9], [10]. As shown in Table 1, the frequency of dark blue plaques, the presence of which indicates an error during lesion bypass, is unchanged when RPA is included in the reaction. The observed value of 33% is the same as previously published values [9]. After sequencing mutant plaques, we calculated an error rate of 5000×10^−4^ for dATP misinsertion, corresponding to roughly equal incorporation of dATP and dCTP. This is the same rate that has been reported multiple times for pol η alone using this assay as well as steady state single nucleotide insertion kinetic assays [6]–[8]. An error rate of 3400×10^−4^ was calculated when a longer substrate was used, and also when adding heat inactivated RPA to the reaction (Table S1). Changes of at least 3-fold are the threshold that we consider different in this assay. These data suggest that in this assay, RPA has no detectable effect on the fidelity of 8-oxoG bypass by human pol η.

**Table 1:** The effect of RPA on the lesion bypass fidelity of truncated pol η.

		Dark Blue Plaque Frequency	Error Rate (10^−4^)
	RPA	8-oxoG	8-oxoG to T
η-511	–	*29%* a	*3500* a
	+	33%	5000

Dark blue reversion frequencies and error rates (10^−4^) from the lesion bypass assay on 45mer templates containing 8-oxoG. Values calculated as previously described in [36]. Error rates result from sequencing of 38 and 22 dark blue plaques, respectively.

a Data in italics previously published in [9].

**Figure 1:**
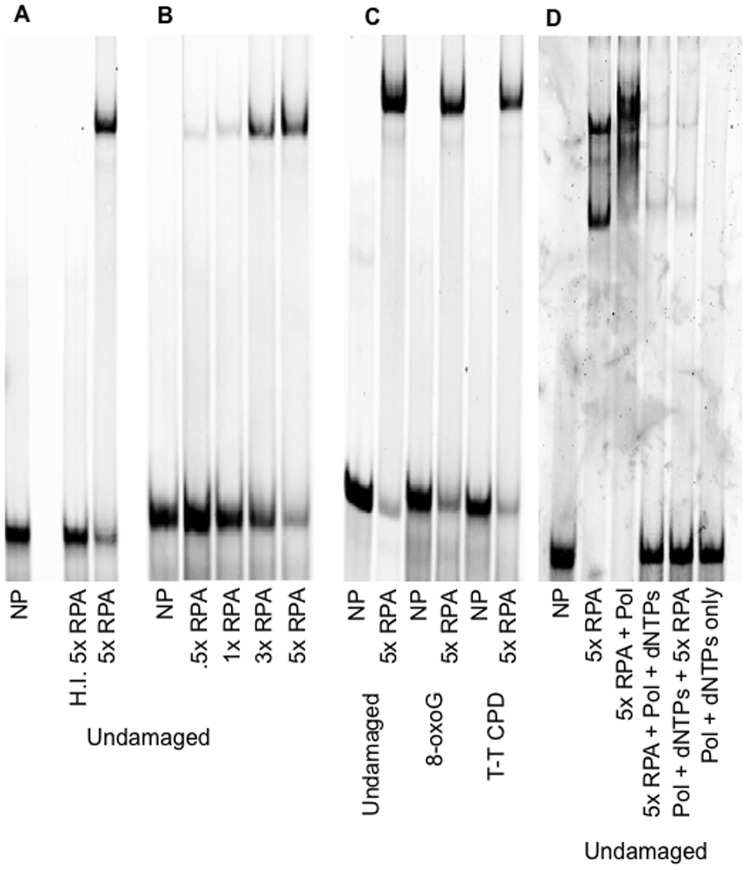
RPA binding to substrates used in the lesion bypass fidelity assay. NP, no protein. HI, heat inactivated (85° for 15 minutes). X RPA indicates fold excess RPA over DNA substrate. Substrates contain 8-oxoG or T-T CPD as indicated. All RPA was bound for 30 minutes at 37° prior to gel separation. **A.** RPA binding to undamaged DNA and heat inactivation of RPA that abolishes binding. **B.** Titration of RPA binding to undamaged DNA. **C.** RPA binding to DNA containing 8-oxoG (substrate shown in Figure 2A) and T-T CPD (substrate shown in Figure 2C). **D.** Consecutive binding of RPA and pol η. RPA is displaced by pol η synthesis. RPA is unable to bind double-stranded DNA after DNA synthesis. All images run on 6% acrylamide:TBE gels. Substrates contained primers 5′ end labeled with Cy5 and imaged on a Storm 865 imager (GE Life Sciences). The upper band in the 5X RPA lane of panel D was is thought to be a DNA-RPA-RPA complex. It was not seen in the gels used in Panels A–C.

**Figure 2:**
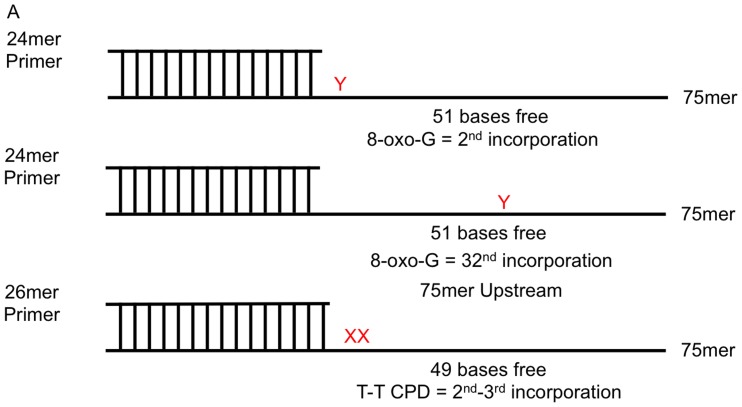
Substrate Diagrams. Schematic diagram of the DNA substrates used in RPA binding and DNA fidelity assays. Y indicates the position of normal guanine or 8-oxoG. 8-oxoG substrates contain a 24-mer primer with the lesion occurring at the 2^nd^ or 32^nd^ incorporation. XX indicates the position of T-T CPD. T-T CPD template contains a 26-mer primer that causes the 3′-T to be the 2^nd^ incorporation after synthesis begins. All templates are 75 bases in length.

Since pol η and RPA have not been reported to physically interact, we hypothesized one way that RPA could affect pol η fidelity is the manner in which RPA binds to DNA. We first confirmed RPA binding to a DNA substrate containing either 49 or 51 bases of single stranded template region using non-denaturing PAGE (Figure 1). Representative substrate diagrams are shown in Figure 2. The substrates used contain either a 24 or 26 base region of primer:template duplex on a 75 base long template, giving either 49 or 51 bases of single stranded region in which RPA can bind, well above its largest binding footprint of ∼30 bases [37]. As seen in Figure 1A, RPA does indeed bind our substrate DNA, and heat inactivation of RPA (85°C, 15 minutes) abolishes this interaction. Increasing amounts of RPA show increased amount of binding (Figure 1B), and we observe that the presence of damage does not seem to change binding to our substrates (Figure 1C). Importantly, RPA and pol η can also bind to the substrate simultaneously, as seen by the supershift present in Figure 1D. Additionally, synthesis by pol η appears to displace RPA from the DNA, evident by the addition of deoxynucleotides to the reaction that shows a near complete abolishment of RPA binding the DNA. Presumably the creation of fully duplex DNA is the cause of this lack of binding.

**Figure 3:**
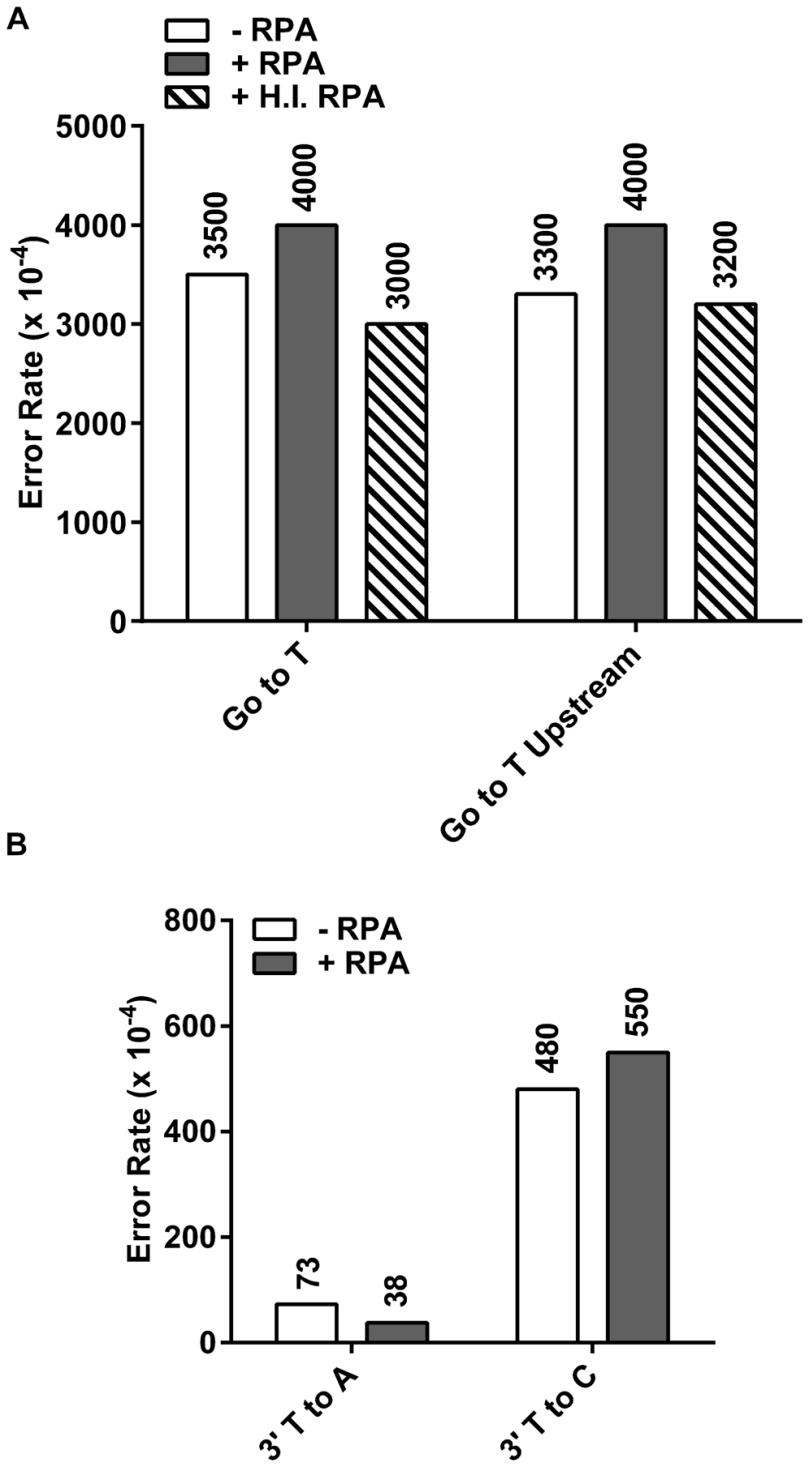
Wild type, full-length η damaged error rates. Data from the lesion bypass fidelity assay as described in Materials and Methods. Substrate to polymerase ratios were 1∶1. Error rates were calculated from sequencing between 23 and 47 dark blue plaques per experiments, with each plaque representing a unique bypass event. White bars indicate the absence of RPA. Dark Grey bars indicate the addition of 5 fold RPA over substrate. Hatched bars indicate the addition of 5 fold heat inactivated RPA over substrate. Heat inactivated RPA created by heating an aliquot of RPA to 85°C for 15 minutes. **A.** 8-oxoG to T (misincorporation of dATP opposite 8-oxoG) error rates (10^−4^) on 75mer and 75mer upstream templates containing 8-oxoG. Values represent average of 2 independent experiments. **B.** 3′-T to C and 3′-T to A errors (misincorporation of dGTP and dTTP, respectively) on 75mer templates containing T-T CPD.

While we cannot definitely say from the gel shift experiments that pol η and RPA physically interact, we recognize the limitations of using a truncated protein that excludes the C-terminal 202 amino acids. Therefore, we then expressed and purified a full-length pol η in an *E. coli* expression system [34] and performed fidelity measurements as described above (examples of all purified proteins can be seen in Figure S1). As can be seen in Figure 3A, the addition of RPA gives error rates the same as when polymerase only was used (3500×10^−4^ and 4000×10^−4^ for polymerase alone and with RPA, respectively). Heat inactivation of RPA once again shows no difference compared to either polymerase alone or functional RPA (error rate of 3000×10^−4^). We also examined if adding RPA was able to alter the fidelity when copying a T-T CPD. Using this assay, it has been previously reported that the most common mutations at the 3′-T are changes to C, with changes to A being less frequent, and to G occurring least frequently [2], [9], [10], [31]. Here, we see that the addition of RPA gives error rates the same as those obtained in its absence (Figure 3B). From these data, we conclude that RPA does not change the fidelity of full-length human pol η when copying either 8-oxoG or a T-T CPD.

Given the striking enhancement of fidelity reported by Maga *et al*, we sought an explanation for how RPA might affect the fidelity of the polymerase when it seems likely that it will have been displaced from the lesion DNA prior to polymerase copying of the lesion. We reasoned that possibly the binding of RPA caused a change in the conformation of the DNA such that the *anti* versus *syn* form was favored for 8-oxoG, allowing more frequent dCTP incorporation. The length of the oligo makes it possible that RPA could be binding the 3′ end of the template strand while pol η is bound at the primer terminus. Since we were unable to use the same sequence as that report (it is incompatible with the *LacZ* based screening we use in this assay), we instead created a substrate with a suitable sequence that contained the 8-oxoG lesion further away from the primer terminus, and somewhere within where RPA is expected to bind the substrate (Figure 2B). This substrate requires synthesis of many more undamaged bases prior to lesion bypass. In this context, the error rates observed for 8-oxoG bypass were 3300×10^−4^ without RPA, 4000×10^−4^ with RPA, and 3200×10^−4^ with heat inactivated RPA (Figure 3B) These numbers are essentially the same as those seen in the “close” position context and also again show that the addition of RPA does not change the fidelity of the polymerase (Figure 3).

We have recently reported on several single amino acid mutants of pol η that display altered fidelity [9], [10]. Given that we failed to see an alteration of fidelity by the addition of RPA, we propose that the main determinant of pol η dependent 8-oxoG bypass fidelity is the properties of the polymerase itself. To test this idea, we generated full-length pol η that contained 2 of the single amino acid substitutions that we have described as having altered fidelity [9]. In the truncated construct, both the Q38A and Y52E mutants show specific changes to fidelity for different errors, depending on the template being copied. If it is only the properties of the polymerase that determine lesion bypass fidelity, we hypothesized that we should see the same altered fidelity signature whether RPA is present or not.

**Figure 4:**
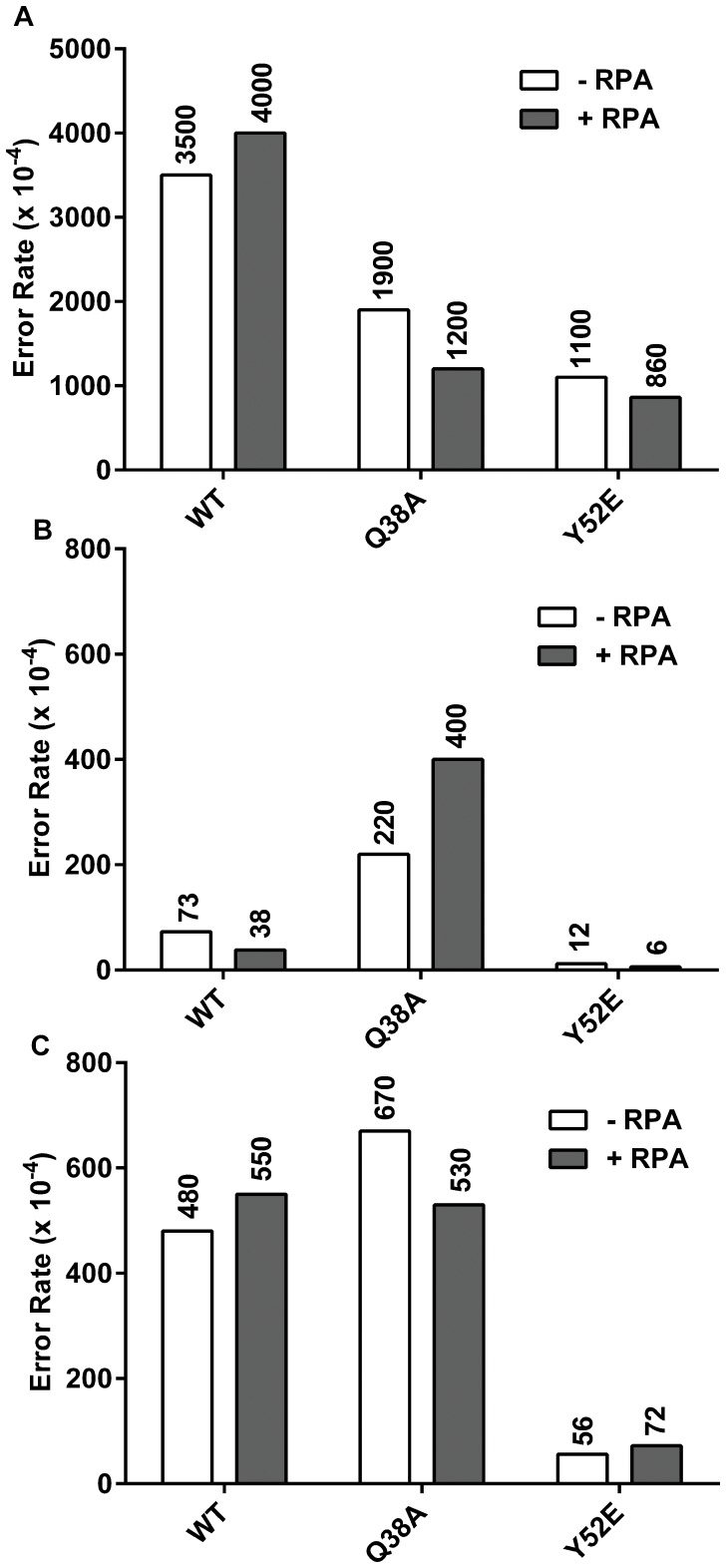
Wild type, Q38A, and Y52E damaged error rates. Data from the lesion bypass fidelity assay as described in Materials and Methods. Error rates were calculated from sequencing between 23 and 47 dark blue plaques per experiment, with each plaque representing a unique bypass event. Substrate to polymerase ratios were 1∶1. White bars indicate the absence of RPA. Dark Grey bars indicate the addition of 5 fold RPA over substrate. **A.** 8-oxoG to T error rates (10^−4^) (misincorporation of dATP opposite 8-oxoG) when copying 75mer templates. **B.** 3′-T to A and **C.** 3′-T to C error rates (10^−4^) (misincorporation of dGTP and dTTP, respectively) when copying 75mer templates containing T-T CPD.

Both Q38A and Y52E showed reduced error rates for the most common 8-oxo-G to T error when compared to wild type polymerase [9]. This effect is recapitulated using the full-length protein (Figure 4A), providing further evidence that the fidelities of full-length and truncated pol η are similar (see also Table S1). Q38A and Y52E display error rates about 3-fold lower than wild type, and the addition of RPA does not change these values. Additionally, the signature of the Q38A mutant of increased 3′-T to A changes compared to wild type when copying a TT dimer is present and maintained in the presence of RPA (Figure 4B). The Y52E mutant shows a reduction in 3′-T to A changes compared to wild type when copying a TT dimer and is also unaffected by the addition of RPA. The reduction of 3′-T to C changes observed in the Y52E mutant is also maintained regardless of RPA status (Figure 4C). Overall, the mutation signatures we published for the truncated mutants hold true for the full-length mutants, and the addition of RPA to the full-length polymerases does not alter these signatures when copying damaged DNA templates.

**Figure 5:**
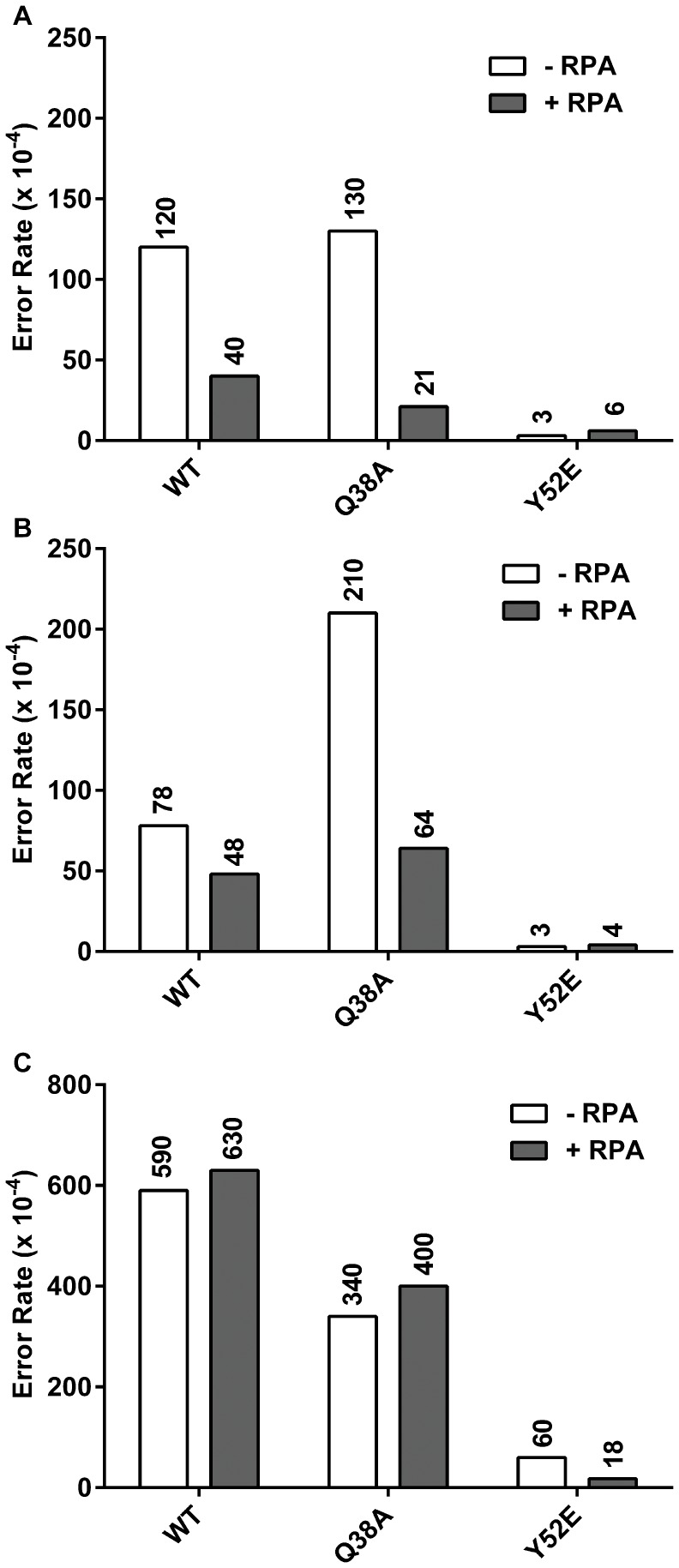
Wild Type, Q38A, and Y52E undamaged error rates. All data from the lesion bypass fidelity assay as described in Materials and Methods. Error rates were calculated from sequencing between 22 and 48 dark blue plaques per experiment, with each plaque representing a unique bypass event. Substrate to polymerase ratios were 1∶1. White bars indicate the absence of RPA. Dark Grey bars indicate the addition of 5 fold RPA over substrate. **A.** G to T error rates (10^−4^) (misincorporation of dATP opposite G) when copying 75mer templates. **B.** 3′-T to A and **C.** 3′-T to C error rates (10^−4^) (misincorporation of dGTP and dTTP, respectively) when copying 75mer undamaged templates.

Pol η also plays a role in copying structures other than base lesions [38]–[43], so we examined the ability of RPA to the alter the fidelity when copying undamaged DNA (Figure 5). Interestingly, there are four instances of at least 3-fold change. For changes at undamaged T to A, we see a reduction in error rate from 210×10^−4^ to 64×10^−4^ when adding RPA to reactions containing the Q38A form of pol η (Figure 5B), a difference of just over 3 fold. A similar 3 fold reduction was seen for T to C changes in the Y52E reaction when adding RPA (60×10^−4^ to 18×10^−4^) (Figure 5C). This same reduction level was seen for wild type when examining undamaged G to T changes when adding RPA (120×10^−4^ to 40×10^−4^) (Figure 5A). For the Q38A mutant, this difference was even more pronounced when examining G to T changes. We observed an over 6 fold reduction, from 130×10^−4^ to 21×10^−4^ for this mutant (Figure 5A). To understand the significance of these changes, we must examine the underlying calculation of these error rates. For the Y52E T to C change, in polymerase only reactions they accounted for 23 of the 36 sequenced plaques (64%), and for 15 out of 30 (50%) when RPA was present. Therefore, we consider this change real and not the result of low sample number. However, when examining Q38A undamaged error rates for T to A and G to T, the picture is more nuanced. For each of these errors, at least 1 change was observed out of a total of 30–32 sequenced mutant plaques but the sample numbers were not as great. For the T to A change, we found 5 of 32 (16%) for polymerase only, and 3 out of 30 (10%) with RPA. For G to T changes, there were 3 out of 32 (9%) for polymerase alone and 1 out of 30 (3%) with RPA. Since these changes (G to T, T to A) are relatively rare events on undamaged DNA, we would need to sequence significantly more samples to achieve adequate numbers of each type to make more definitive statements on the significance of these changes. The G to T change with wild type polymerase was observed in 8 of 48 sequenced samples (17%), so we consider the change real but also note that the amount of change falls right at the 3-fold cutoff.

While some of the observed differences when RPA is added may be a result of low numbers of observed mutants, we also see interesting patterns for the Q38A mutant when examining two other types of changes. Due to slight translational read through of the stop codon in the LacZ sequence used in this assay, frameshift errors at the stop codon are detectable as they result in true colorless plaques, rather than light blue (and opposed to the dark blue of true reversion mutations) [44]. When examining the colorless plaque frequency for Q38A experiments, the colorless frequency is reduced ∼3 fold with the addition of RPA when examining 8-oxoG and undamaged templates, but remains similar when using T-T CPD substrates (Figure S2). Another advantage of utilizing an assay that requires both insertions across from the damaged nucleotides as well as extension beyond the lesion is the ability to detect complex changes, such as tandem base substitutions, which pol η is known to make [4], [5], [9], [10], [31]. When looking at complex error rates, we note that Q38A again shows a similar pattern to that of frameshift errors. Complex errors at 8-oxoG are decreased greater than 7 fold with the addition of RPA and are decreased slightly more than 3 fold with the addition of RPA at undamaged bases. This reduction is not seen for complex changes when examining T-T CPD (Figure S3). The glutamine residue at position 38 in pol η contacts the template base in the active site of the protein [45], and abolishment of this interaction by substitution with alanine increases the number of complex errors seen when copying undamaged DNA [9]. It is possible that RPA binding to DNA somehow stabilizes the template DNA in the active site for this mutant polymerase, reducing the occurrence of these errors when copying 8-oxoG and undamaged DNA. This stabilization may not be evident with wild type pol η due to intact template contact [45] or with a T-T CPD, which has two templates bases physically crosslinked [46]. This is reminiscent of effects seen on mutant polymerases of the bacteriophage RB69 [18].

This reduction in error rate, if real, could play a role in reducing the numbers of errors introduced when copying undamaged DNA, for example when pol η is involved in DNA copying during somatic mutation of immunoglobulin genes, homologous recombination, or copying of other complex undamaged DNA structures. We find it interesting that a similar reduction in error rate specifically when copying undamaged DNA has been previously reported for single amino acid substitution mutants in the little finger region of pol η [10] and a reduction in undamaged DNA error rate was also observed in other active site mutants [9]. Combined, these results suggest that the error rate of TLS by wild type pol η, at least for 8-oxoG and a T-T CPD, is controlled largely by the structure of the polymerase active site. Mutations to the polymerase that can affect fidelity do so in large part by changing the fidelity on undamaged DNA synthesis while leaving the fidelity of TLS largely untouched. We maintain that the low-to-moderate fidelity of pol η mediated TLS does indeed represent a tradeoff between the risk of introducing base substitution or small frameshift mutations and the risk of having a stalled replication fork and/or un-replicated stretches of DNA that can lead to aberrant (and much more likely to be mutagenic) processing, like non-homologous end joining.

An unresolved issue we have not yet addressed regarding the ability of replication accessory proteins to affect TLS fidelity is the role of PCNA. In Maga *et al*, they tested both RPA and PCNA in combination [7]. McCulloch *et al* also tested the combination of RPA, PCNA, and the clamp loading complex RFC using yeast proteins [8]. While McCulloch *et al* reported no change in fidelity, it is still possible that in the human system PCNA will play a role in altering fidelity. To these ends, it will be interesting to see if the combination of RPA and PCNA has a combined effect on human pol η, and also whether unmodified and mono-ubiquitylated PCNA have different properties in this regard.

## Supporting Information

Figure S1 **Sypro-Red stained protein gels of purified proteins.** Sizes are indicated for relevant marker bands. All gels imaged with a Storm 865 imager (GE Life Sciences). **A.** C-terminal 6x-His tagged truncated pol η (1–511 aa) separated by 10% SDS-PAGE Gel. **B.** Purification overview of N-Terminal 6x-His tagged pol η produced in *E. coli*. Fractions represent samples taken after centrifugation, pool after the nickel column, pool after the hydroxyapatite column, individual fractions from the mono s column, and the pooled result as stored. Samples separated by 10% SDS-PAGE. **C.** Final pools of RPA separated by 4–20% SDS-PAGE. 3 subunits of RPA (p70, p32, p14) marked as indicated.
(TIF)

Figure S2 **Colorless plaque frequency.** All data from the lesion bypass fidelity assay as described in Material and Methods. Percentage of colorless plaques of the whole number of plaques counted. Total plaques counted between 1,000 and 20,000 per condition. White bars indicate the absence of RPA. Dark Grey bars indicate the addition of 5 fold RPA over substrate. Hatched bars indicate the addition of 5 fold heat inactivated RPA over substrate.
(TIF)

Figure S3 **Complex error rate.** All data from the lesion bypass fidelity assay as described in Material and Methods. Complex errors defined as any changes at multiple bases with less than 2 correct insertions in between. Examples include 2 sequential base substitutions (majority), base substitution followed by correct insertion followed by another base substitution, or multiple base deletions. Numbers with an (*) indicate no mutant plaques sequenced contained a complex change and the error rate is displayed as a maximal possible rate (calculated based on the value if there were 1 observed change). White bars indicate the absence of RPA. Dark Grey bars indicate the addition of 5 fold RPA over substrate. Hatched bars indicate the addition of 5 fold heat inactivated RPA over substrate. All values are given as errors per 10,000 insertion events (i.e.×10^−4^).
(TIF)

Table S1 **Truncated and full-length pol η comparison.** Dark blue plaque frequencies and error rates (10^−4^) from lesion bypass fidelity assay on 75mer templates. Templates contained 8-oxoG. H.I. indicates addition of heat inactivated RPA (85°C for 15 minutes). Values for full-length represent the average of 2 independent experiments. Values calculated as previously described in [36]. Error rates result from sequencing between 23 and 47 dark blue plaques.
(DOCX)
