# Supplementary figures and images for: Biochemical Analysis of DNA Polymerase η Fidelity in the Presence of Replication Protein A
Source: PLoS One. 2014 May 13;9(5):e97382. doi: 10.1371/journal.pone.0097382 (PMC4019591; doi:10.1371/journal.pone.0097382)

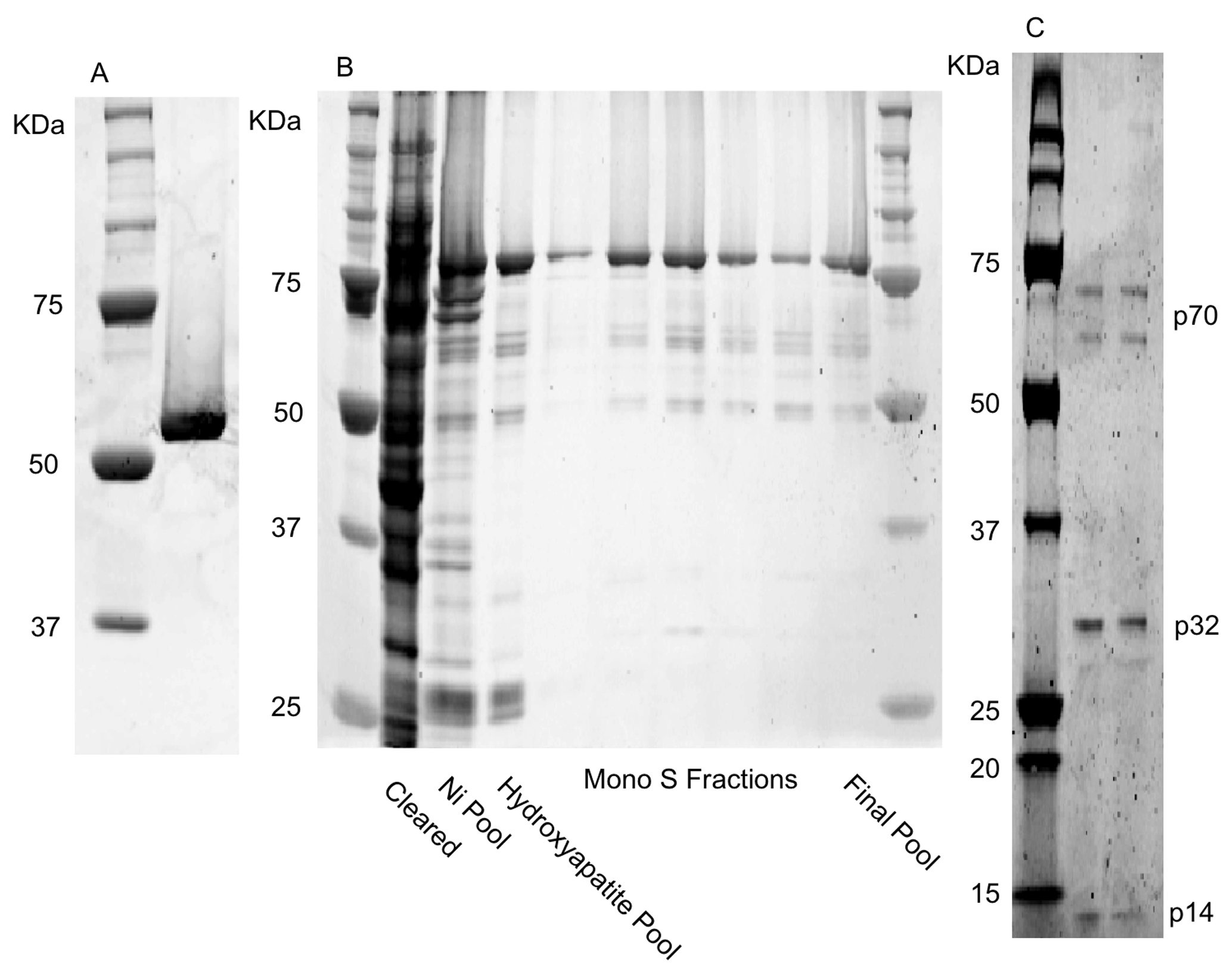

Supplement: Figure S1 — Sypro-Red stained protein gels of purified proteins. Sizes are indicated for relevant marker bands. All gels imaged with a Storm 865 imager (GE Life Sciences). A. C-terminal 6x-His tagged truncated pol η (1–511 aa) separated by 10% SDS-PAGE Gel. B. Purification overview of N-Terminal 6x-His tagged pol η produced in E. coli. Fractions represent samples taken after centrifugation, pool after the nickel column, pool after the hydroxyapatite column, individual fractions from the mono s column, and the pooled result as stored. Samples separated by 10% SDS-PAGE. C. Final pools of RPA separated by 4–20% SDS-PAGE. 3 subunits of RPA (p70, p32, p14) marked as indicated. (TIF) [file pone.0097382.s001.tif]

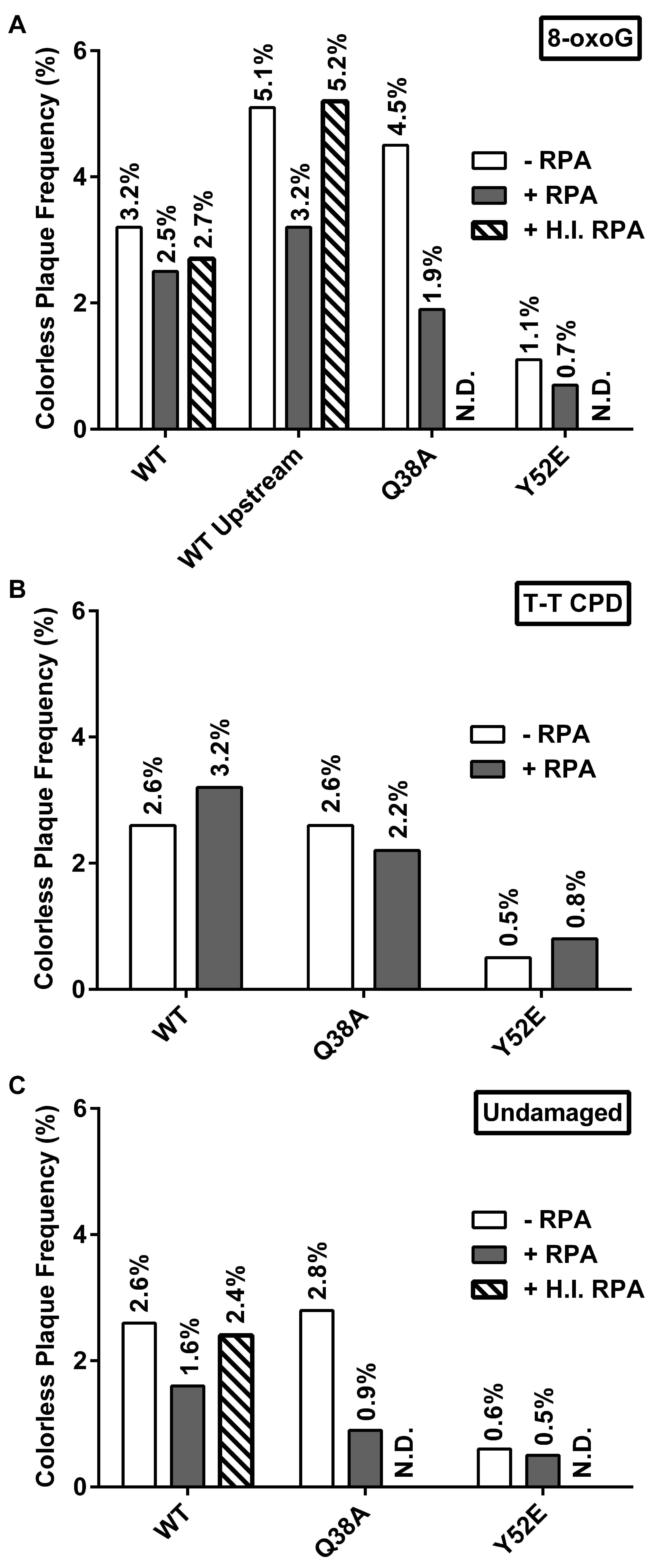

Supplement: Figure S2 — Colorless plaque frequency. All data from the lesion bypass fidelity assay as described in Material and Methods. Percentage of colorless plaques of the whole number of plaques counted. Total plaques counted between 1,000 and 20,000 per condition. White bars indicate the absence of RPA. Dark Grey bars indicate the addition of 5 fold RPA over substrate. Hatched bars indicate the addition of 5 fold heat inactivated RPA over substrate. (TIF) [file pone.0097382.s002.tif]

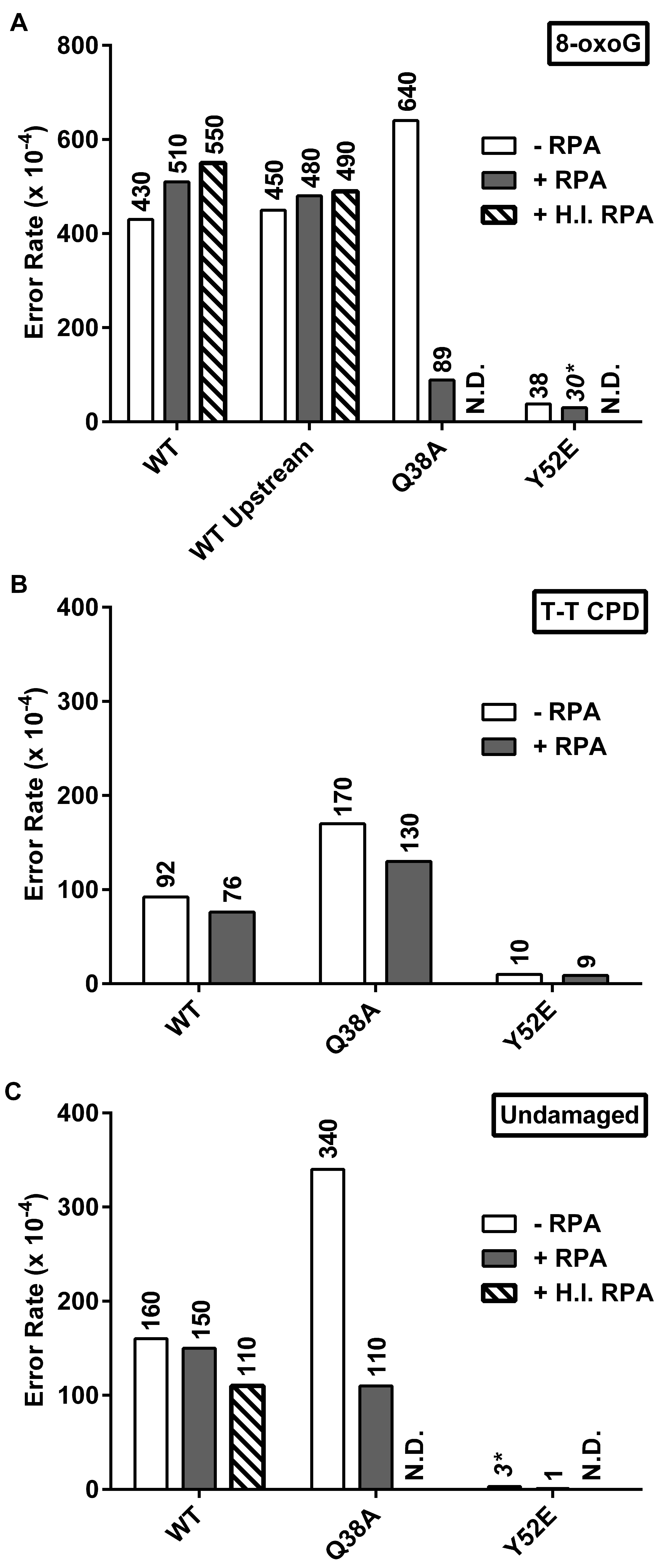

Supplement: Figure S3 — Complex error rate. All data from the lesion bypass fidelity assay as described in Material and Methods. Complex errors defined as any changes at multiple bases with less than 2 correct insertions in between. Examples include 2 sequential base substitutions (majority), base substitution followed by correct insertion followed by another base substitution, or multiple base deletions. Numbers with an (*) indicate no mutant plaques sequenced contained a complex change and the error rate is displayed as a maximal possible rate (calculated based on the value if there were 1 observed change). White bars indicate the absence of RPA. Dark Grey bars indicate the addition of 5 fold RPA over substrate. Hatched bars indicate the addition of 5 fold heat inactivated RPA over substrate. All values are given as errors per 10,000 insertion events (i.e.×10−4). (TIF) [file pone.0097382.s003.tif]
