# Supplementary material for: Biochemical Analysis of DNA Polymerase η Fidelity in the Presence of Replication Protein A
Source: PLoS One. 2014 May 13;9(5):e97382. doi: 10.1371/journal.pone.0097382 (PMC4019591; doi:10.1371/journal.pone.0097382)
Supplement: Table S1 — Truncated and full-length pol η comparison. Dark blue plaque frequencies and error rates (10−4) from lesion bypass fidelity assay on 75mer templates. Templates contained 8-oxoG. H.I. indicates addition of heat inactivated RPA (85°C for 15 minutes). Values for full-length represent the average of 2 independent experiments. Values calculated as previously described in [36]. Error rates result from sequencing between 23 and 47 dark blue plaques. (DOCX) [file pone.0097382.s004.docx]

**Table S1**

|  |  | *Dark Blue Plaque Frequency* | *Error Rate (10^-4^)* |
| --- | --- | --- | --- |
|  | **RPA** | **8-oxoG** | **8-oxoG to T** |
| **η-511** | -^b^ | *29%* ^a^ | *3500* ^a^ |
|  | +^b^ | 33% | 5000 |
|  | + | 25% | 3400 |
|  | H.I. | 23% | 3400 |
| **η-713** | - | 28% | 3500 |
|  | + | 32% | 4000 |
|  | H.I. | 28% | 3000 |

^a^ Data in italics previously published in [9].

^b^ Data collect on a 45mer template
